# Genial tubercle position and genioglossus advancement in obstructive sleep apnea (OSA) treatment: a systematic review

**DOI:** 10.1186/s40902-019-0217-1

**Published:** 2019-09-09

**Authors:** Edward T. Chang, Yong-Dae Kwon, Junho Jung, Robson Capasso, Robert Riley, Stanley C. Liu, Macario Camacho

**Affiliations:** 1 Department of Otolaryngology, Division of Sleep Surgery and Medicine, Tripler Army Medical Center, Honolulu, HI USA; 2 Department of Oral and Maxillofacial Surgery, Kyung Hee University School of Dentistry, Seoul, Republic of Korea; 3 Department of Otolaryngology - Head and Neck Surgery, Sleep Surgery Division, Stanford University Medical Center, Stanford, CA USA; 4 Department of Psychiatry and Behavioral Sciences, Division of Sleep Medicine, Stanford Hospital and Clinics, Redwood City, CA USA

**Keywords:** Obstructive sleep apnea, Genioglossus advancement, Skeletal surgery, Genioglossus muscle, Mandible

## Abstract

**Background:**

To systematically review the literature for methods to localize the genial tubercle as a means for performing an advancement of the genioglossus muscle.

**Methods:**

PubMed, Google Scholar, CRISP, EMBASE, CINAHL, and Scopus were searched from inception through June 16, 2015.

**Results:**

One hundred fifty-two articles were screened, and the full text versions of 12 articles were reviewed in their entirety and 7 publications reporting their methodology for localizing the genial tubercle. Based upon these measurements and the results published from radiographic imaging and cadaveric dissections of all the papers included in this study, we identified the genial tubercle as being positioned within the mandible at a point 10 mm from the incisor apex and 10 mm from the lower mandibular border.

**Conclusion:**

Based upon the results of this review, the genial tubercles were positioned within the mandible at a point 10 mm from the incisor apex and 10 mm from the lower mandible border. It may serve as an additional reference for localizing the genial tubercle and the attachment of the genioglossus muscle to the mandible, although the preoperative radiological evaluation and the palpation of the GT are recommended to accurately isolate.

## Background

Obstructive sleep apnea (OSA) poses a significant medical problem plaguing a diverse group of individuals within all demographics throughout the world. USA Today reports an increase in cases of OSA within the US Military community by 150% from 2009 to 2013. Genioglossus advancement (GA) surgery effectively addresses the problem of retrolingual airway narrowing with minimal surgical intervention. Many surgeons balk at this surgical modality due to difficulty in isolating the position of the genioglossus muscle and increased risk of devitalizing teeth. We present an additional reference to localize the genial tubercle for increasing ease of the genioglossus advancement procedure based on a systematic review of pertinent literature on this topic from1984 to 2013.

Per its definition, repetitive episodes of pharyngeal collapse with increased airflow resistance leads to obstructive sleep apnea. Treatment and management of OSA necessitates various medical and surgical interventions. Selection of the appropriate treatment modality relies significantly upon the degree and type of obstructive pathology [1–4]. Surgical alternatives remain reserved for treatment of the most severe forms of OSA and patients with poor compliance on continuous positive airway pressure (CPAP) devices [4, 5].

Among various surgical methods, genioglossus advancement provides an effective technique to resolve the obstructive pathology caused by retrolingual airway narrowing. Riley et al. first described the GA technique for the treatment of OSA in 1984 [6]. Since its inception, several studies validated the utility of this technique [7, 8]. When performed correctly, the patient enjoys distinct improvement in retrolingual airway patency. However, the key to ensuring success of this surgical treatment requires accurate assessment and localization of the attachment of the belly of the genioglossus muscle to the mandible to ensure maximal advancement of the genioglossus muscle [8].

In order to identify the position of the genioglossus muscle, the surgeon employs various methods to include manual palpation and more recently, cone beam computed tomography (CBCT). However, the surgeon typically encounters significant difficulty in accurately identifying the position of the genioglossus muscle and its point of attachment to the mandible. Consequently, in the absence of surgical dissection to directly visualize the actual bony attachment of the muscle, the GA technique requires some degree of “blind luck” in identifying the primary point of attachment of the belly of the genioglossus muscle to the mandible [6–8].

Realizing the importance of isolating and identifying the attachment of the genioglossus muscle to the mandible, we conducted an exhaustive systematic review of the literature from 1984 to 2013. These studies provided measurements correlated with cephalometrics identifying the position of the genioglossus muscle and its attachment to the mandible. From a systematic review of the measurements from these articles, we developed the Rule of Tens with respect to the genial tubercle in assisting the surgeon to more accurately localize the attachment of the genioglossus muscle to the mandible and further enhance efficacy of the GA surgical technique.

## Methods

We conducted a systematic review of all literature written in or translated into English from 1984 to March 2015 on the topic of Genioglossus Advancement. The search revealed articles with topics that included measurements collected from cadaveric and/or radiographic studies. MEDLINE served as the primary database of the majority of the literature search. In addition, each author also conducted individual searches on PubMed, CRISP, EMBASE, CINAHL and Scopus. The systematic search used the following search terms: “geniotomy” OR “genioglossus advancement” OR “genial tubercle” OR “genial tubercle advancement” OR “genioglossus muscle advancement” OR “GTA” OR “GGA” OR “inferior sagittal mandibular osteotomy” OR “mandibular osteotomy” OR “sliding genioplasty” AND “sleep.” The result of the literature search and review identified more than one hundred articles on these topics. Of these, seven articles met inclusion criteria as assessed by the authors (YDK, ETC and MC) who individually reviewed the abstracts of all searched articles for information pertinent to this systematic review.

Upon final review, the authors excluded two articles written in Chinese due to the absence of English translation for the remainder of the papers and possible discrepancy in content. One article in particular appeared to present pertinent information likely to contribute to this systematic review; however, the lack of English translation hindered an accurate review and assessment [9]. The authors included the remaining five articles following a comprehensive review of the entire text in English [10–14]. To further identify possible relevant articles, the authors reviewed the references cited in each individual article. The authors identified two potential papers. However, upon final evaluation, the authors excluded these identified articles due to age and lack of relevance [15, 16]. In addition, the authors of one paper adopted a conventional tomography technique which appeared to lack validity and fraught with inconsistent images [15].

## Results

**Fig. 1:**
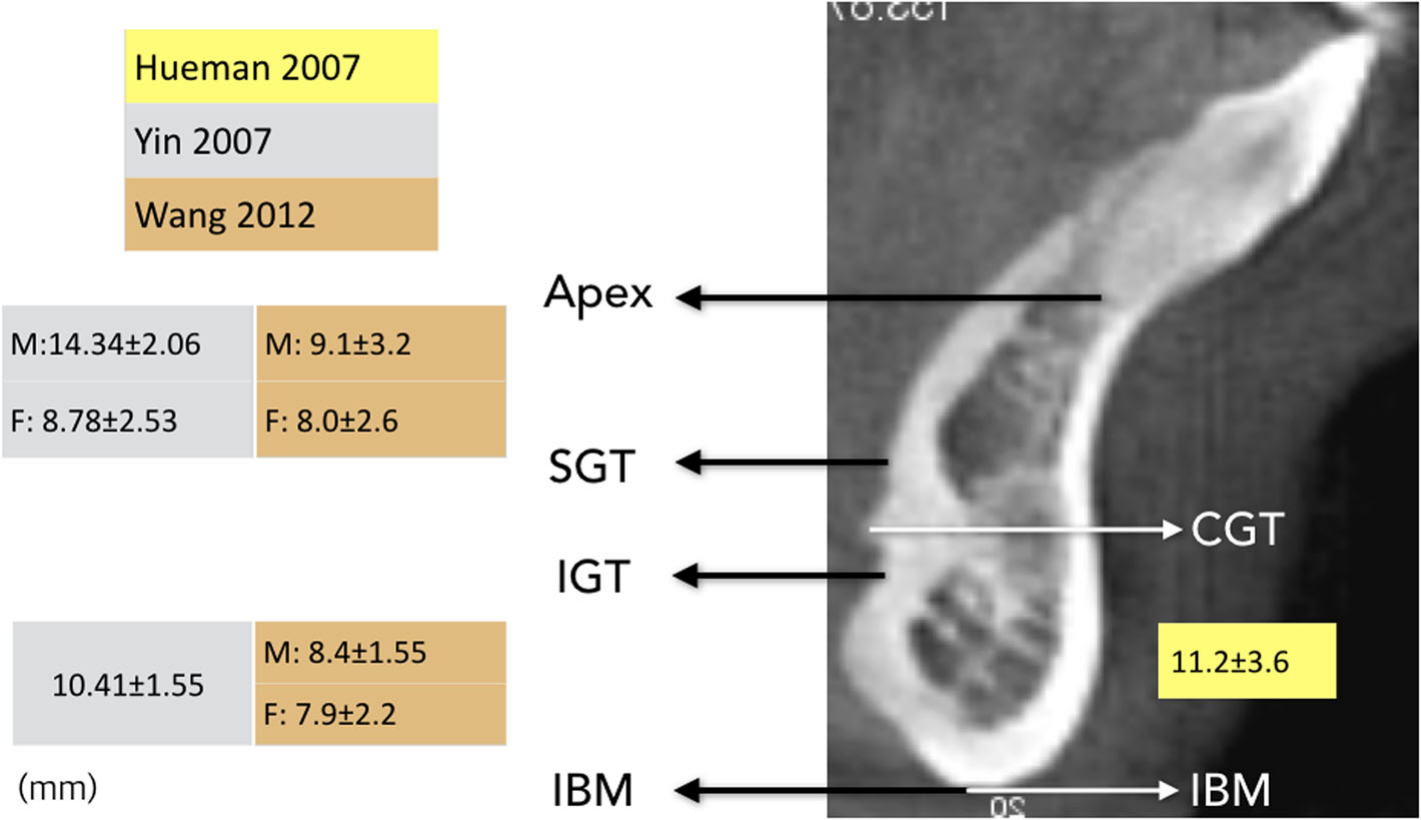
Position of the genial tubercle evaluated by radiographic measures

Figure 1, Table 1, and Table 2 provide a list of the selected and comprehensively reviewed articles. Two articles described cadaveric studies and one study provided a description based on a radiographic assessment. The remaining two articles presented validation studies comparing CT scans with the measurements obtained from cadaveric dissections. Due to the inherent heterogenicity among the identified references, direct comparisons of the pertinent measured anatomical landmarks and the specific measurement points remained difficult.

**Table 1:** Comparison of the measurements for the genial tubercle position evaluated by radiographic measures

Literatures	Radiographic study	*N*	IBM–IGT	IBM–CGT	Apex–SGT	GTH	GTW	MT (IGT)	MT (CGT)
Hueman 2007	CBCT	17	NA	11.2 ± 3.6	NA	5.1 ± 1.6	5.3 ± 1.2	14.4 ± 2.8	NA
Yin 2007	SPIRAL	40 (29/11)	10.41 ± 1.55	NA	M: 14.34 ± 2.06	6.17 ± 0.71	7.01 ± 1.13	12.9 ± 1.64	NA
			ND	ND	F: 8.78 ± 2.53	ND	ND	ND	NA
Wang 2012	CBCT	48 (23/25)	M: 8.4 ± 1.55		M: 9.1 ± 3.2	M: 6.5 ± 1.7	M: 7.8 ± 1.6	NA	M: 13.3 ± 1.7
			F: 7.9 ± 2.2		F: 8 ± 2.6	F: 6.7 ± 1.9	F: 7.1 ± 1.6	NA	F: 12.7 ± 2.1

*IBM–IGT* distance from the inferior border of the genial tubercle (IGT) to the inferior border of the mandible (IBM), *Apex–SGT* distance from the root apex of the mandibular incisor tooth (Apex) to the superior border of the genial tubercle (SGT), *GTH* genial tubercle height which is the distance from the superior border of the genial tubercle (SGT) to the inferior border of the genial tubercle (IGT), *GTW* genial tubercle width, *MT* thickness of the anterior mandible

**Table 2:** Comparison of the measurements for the genial tubercle position evaluated from cadavers

Literatures	Cadaveric	*N*	IBM–IGT	IBM–CGT	Apex–SGT	GTH	GTW	MT (IGT)	MT (CGT)
Silverstein 2000		10	NA	14.2 ± 2.2	11.8 ± 1.69	NA	NA	NA	12.6 ± 1.84
Hennessee 2005		7	12.5 ± 1.44	NA	NA	11.64 ± 1.84	6.86 ± 1.03	NA	NA
Hueman 2007		17	NA	13.3 ± 2.9	NA	4.7 ± 1.5	4.9 ± 0.9	14.3 ± 2.2	NA
Yin 2007		40 (29/11)	11.08 ± 2.05	NA	M: 15.57 ± 1.82	5.82 ± 0.71	6.98 ± 1.35	11.95 ± 1.59	NA
					F: 9.36 ± 2.79				

*IBM–IGT* distance from the inferior border of the genial tubercle (IGT) to the inferior border of the mandible (IBM), *IBM-CGT* distance from the center of the genial tubercle (CGT) to the inferior border of the mandible (IBM), *Apex–SGT* distance from the root apex of the mandibular incisor tooth (Apex) to the superior border of the genial tubercle (SGT), *GTH* genial tubercle height which is the distance from the superior border of the genial tubercle (SGT) to the inferior border of the genial tubercle (IGT), *GTW* genial tubercle width, *MT* thickness of the anterior mandible

The majority of the reviewed literature provided a range for the genial tubercle height (GTH). The data from the radiographic measurements revealed a GTH from 5 to 8 mm. However, one cadaveric study refuted these findings somewhat due to a significant different result in their measurement range for the GTH [10]. Wang et al. [14] presented more detailed radiographic measurements according to the gender and dental occlusion. They measured the distance from the inferior border of the mandible (IBM) to the inferior border of genial tubercle (IGT) (IGT-IBM) as 6 to 11 mm. Of note, other studies measured this distance from the center of the genial tubercle.

Further, Wang et al. provided measurements for the distance from the apices of the lower incisors (LI) to the superior border of the genial tubercle (SGT) (LI-SGT) as 8 to 15 mm. They measured the GTH as 5 to 11 mm, genial tubercle width (GTW) as 5 to 8 mm, and the thickness of the anterior mandible (MT) as 11 to 14 mm. Based upon these measurements and the results published from radiographic imaging and cadaveric dissections of all the papers included in this study, we identified the GT as positioned within the mandible at a point 10 mm from the incisor apex and 10 mm from the lower mandible border. However, due to the individual anatomical variations of the chin, the height of the GT cannot be concluded with the position. Therefore, localization of the GT for attachment of the bellies of the genioglossus muscle should be preoperatively evaluated using imaging modalities.

## Discussion

Since the introduction of the GA procedure for OSA patients as an effective method of mandibular inferior border osteotomy for genioglossus advancement [6, 7], surgeons attempted other variations on this technique to improve efficacy. Currently, the most widely utilized technique employs the formation of a rectangular osteotomy within the mandible. When performed correctly, the GA surgical procedure addresses the retrolingual obstruction and significantly improves patency of the airway [5–8].

The success or failure of the GA technique relies prominently in accurately identifying the attachment of the belly of the genioglossus muscle to the mandible. From anatomical studies based on cadaveric dissections and radiographic imaging, the skeletal attachment of the genioglossus muscle appears localized about the genial tubercle. As with many anatomical structures, some degree of variation exists among the patient population.

Moreover, the accurate location of the muscle attachment remains difficult to confirm even intraoperatively causing some degree of consternation to the novice surgeon. Though the genioglossus muscle and genial tubercle area are palpable by means of digital pressure on the anterior mouth floor area, the spatial information and relationship to other anatomical structures remain elusive to the surgeon with limited experience in performing this procedure. Due to this inherent uncertainty and difficulty in localizing the attachment of the genioglossus muscle to the mandible, several groups attempted cadaveric studies to more clearly delineate the skeletal attachment [10, 12, 13].

Other groups augmented these findings with radiographic imaging modalities. Though more practical, imaging based on X-ray radiography presents some limitations on accurately identifying soft tissue structures. Consequently, two studies attempted to validate the radiographic evaluations by comparing cadaveric measurements to those obtained from CT scans [11, 13].

Because OSA typically presents with greater prevalence in skeletal occlusion class II patients with a posteriorly displaced and small chin, the dimension and the location of the genial tubercle appears more important for optimizing the assessment and plan for treatment. One radiographic study using CBCT compared the measurements based on the skeletal pattern and genders [14]. This group’s results revealed that the IGT-IMB (distance from the mandibular inferior border to the inferior margin of genial tubercle) of skeletal class II males was significantly different when compared to that of the skeletal class I female. However, they concluded that no significant differences existed among the skeletal patterns and genders even with the noted difference in IGT-IMB.

As for the dimensional difference between males and females in the context of the local anatomy for genioglossus advancement, most of the literature provided no specificity of the gender of their respective subjects. They did, however, present the distances between the superior border of the genial tubercle and the apex of the lower incisor separately with respect to the gender of the subject [13]. Use of three-dimensional CBCT scans improves visualization of the local anatomy. However, X-ray radiography remains limited in its ability to identify soft tissue morphology. Subsequently, accurate identification of the attachment of the belly of the genioglossus muscle to the mandible during genioglossus advancement remains difficult. Through experience and development of knowledge regarding spatial anatomy, the surgeon gains more expertise in more accurately performing the GA technique.

Previous cadaveric studies provided clear visualization of the genioglossus muscle and its subsequent attachments to the mandible. These studies helped to elucidate a relationship with these attachments to the position of the genial tubercle. To augment this estimation of muscle attachment, imaging modalities rely heavily upon X-ray radiography. Further, in order to validate radiographic studies of the lingual topography of the anterior mandible, some researchers correlated the cadaveric dissection with CT scans. These measurements vary among studies. We reviewed these anatomic and radiographic studies and compared the measurements from each study. From this systematic review, manual palpation to identify genial tubercle might be important; however, with the progress of digital technology and three-dimensional imaging techniques, we can definitely take advantage of the virtual surgery system to capture the genial tubercle [17]. Therefore, based on this observation, individual planning should be considered when genioglossus advancement is planned.

## Conclusion

Based upon the results of this review, the genial tubercles were positioned within the mandible at a point 10 mm from the incisor apex and 10 mm from the lower mandible border. It may serve as an additional reference for localizing the genial tubercle and the attachment of the genioglossus muscle to the mandible, although the preoperative radiologic evaluation and individualized computer-assisted surgery can be further utilized.

## Data Availability

Not applicable

## Abbreviations

CBCT: Cone beam computed tomography
CPAP: Continuous positive airway pressure
GA: Genioglossus advancement
GTH: Genial tubercle height
GTW: Genial tubercle width
IBM: Inferior border of the mandible
IGT: Inferior border of genial tubercle
LI: Lower incisors
MT: Thickness of the anterior mandible
OSA: Obstructive sleep apnea
SGT: Superior border of the genial tubercle
